# Mesentery stiffness changes in a patient with encapsulated peritoneal sclerosis by real-time shear-wave elastography ultrasound with histological reference

**DOI:** 10.1080/0886022X.2023.2183042

**Published:** 2023-03-01

**Authors:** Yutong Zhang, Ming Xu, Xiaoyan Xie, Yujun Chen

**Affiliations:** Department of Ultrasound, The First Affiliated Hospital of Sun Yat-Sen University, Guangzhou, China

Dear Editor,

Long-term ambulatory peritoneal dialysis (PD) in patients with chronic renal failure can lead to encapsulated peritoneal sclerosis (EPS), which is difficult to diagnose and has an estimated incidence of 0.7%; it is characterized by vague symptoms and signs, and mostly manifests as subacute intestinal obstruction such as abdominal pain and nausea, and has a poor prognosis, high mortality, and severe impact on patients’ quality of life [1,2].

The gold standard for EPS diagnosis is surgical exploration and sampling for surgical specimens, however, this method lacks practicability for routine follow-up. EPS diagnosis is based on clinical symptoms, laboratory examination, and computerized tomography (CT). Key CT features for EPS diagnosis are mesenteric and peritoneal thickening, calcification, bowel wall thickening adhesions, and intestinal obstruction, among others [3]. But this method involves exposure to radiation, and in cases where contrast agents are used, the burden on the patient’s kidney increases. Therefore, it is not suitable as a routine method for a series of follow-ups. Ultrasound is an easily accessible, cost-effective, and radiation-free imaging modality for abdominal examinations. The appearance of hyperechoic clumps of small-bowel loops and ‘trilaminar’ membrane has been described as characteristic of EPS [3], but the available information is still limited.

Real-time Shear Wave Elastography (SWE) ultrasound, as an emerging examination method, can provide useful quantitative information on the stiffness of tissues. Pathophysiological changes caused by long-term PD may cause changes in mesenteric stiffness. To the best of our knowledge, the application of SWE for assessing the mesentery to discuss its relationship with EPS has not been explored before.

Herein, we explored the novel method of SWE imaging in PD patients to analyze the association of mesentery stiffness in patients with ambulate PD.

A 63-year-old male patient diagnosed with stage 5 chronic kidney disease caused by chronic glomerulonephritis came to our hospital for the first time in July 2008. He had been receiving PD (1.5% PD solution 2 L*2 bags, 2.5% PD solution 2 L*2 bags) regularly in our hospital for 11 years, with a 700 mL daily excess and +250 mL ultrafiltration. The patient came to the hospital this time (June 2022) complaining of nausea and abdominal pain for 5 days and a loss of 5.7 kg in body weight. Blood urea nitrogen was 13.7 mmol/L, serum creatinine was 793 µmol/L, and CRP was increased to 20.51 mg/L. Peritoneal transport remained low and abdominal computerized tomography (CT) showed multiple calcifications of the peritoneum and mesentery.

SuperSonic Shear Wave EXPLORER (SuperSonic Imagine S.A., Ax-EN-Provence, France) was used for conventional two-dimensional peritoneal ultrasound with SL10-2 linear array probe and 2–10 MHz frequency. Abdominal examination results showed extensive intestinal dilation with wall thickening and serous layer calcification and increased mesenteric echo with calcification, which were pathologic changes caused by long-term PD. There was also a small amount of encapsulated fluid in the pelvic cavity.

SWE ultrasound machines and probes are the same as conventional two-dimensional ultrasound and use elastic functions. Target sites for elasticity measurement were the right lower abdominal mesentery near the ileum terminal intestinal wall. Three measurements were obtained and the mean SWE values were calculated. SWE ultrasound results revealed that the elastic modulus of the mesentery was 20.6 kPa.

In consideration of early EPS, a biopsy was performed 3 days after SWE ultrasound. On microscopy, peritoneum collagen fibers were hyperplastic, vascular walls were thickened and hardened, and mesenchymal cells were exfoliated in the biopsy specimen. Pathological results confirmed EPS diagnosis.

Figure 1 shows abdominal CT, two-dimensional ultrasound, SWE, and histological images.

**Figure 1.**
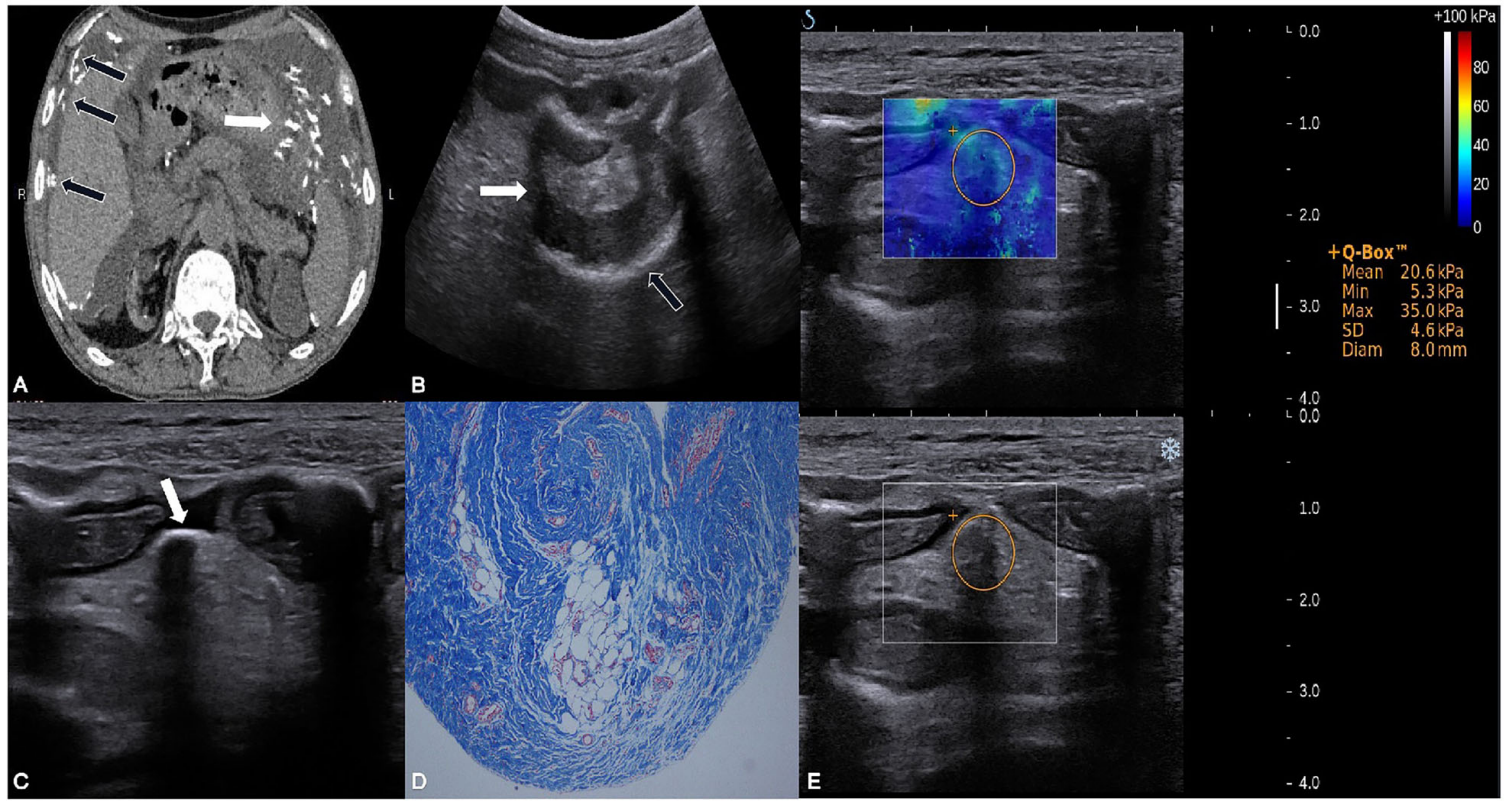
Imaging manifestations of the patient: (A) Abdominal CT image showed multiple calcifications in the parietal peritoneum (black arrows) and mesentery (white arrow), and peritoneal effusion in the patients with EPS. (B–C) Conventional ultrasound image: (B) Intestinal dilation (white arrows) and calcification of serious layer (black arrows). (C) Calcification of the mesentery (white arrow). (D) High-magnification histological image (Masson stain), showed that peritoneum collagen fibers were hyperplasic, vascular walls were thickened and hardened, and mesenchymal cells were exfoliated. (E) Real-time shear-wave elastography (SWE) image of the mesentery overlaying conventional ultrasound grayscale images in the patient with EPS. Circular regions of interest are depicted. The color scale indicates the distribution of the measured elasticity within the circular region of interest. The mean SWE value of mesentery in the patient with EPS was 20.6 kPa.

The patient’s abdominal pain was relieved without intestinal obstruction after modifying the dialysis program to hemodialysis treatment. The patient shows no special symptoms at present.

This report provides the images and characteristics of the patient with long-term ambulatory PD using conventional ultrasound and SWE. EPS Ultrasound findings revealed intestinal wall thickening and calcification, peritoneum thickening and calcification, encapsulated ascites, intestinal dilatation, and increased intestinal wall stiffness. We suggest that the above ultrasound findings, combined with the corresponding clinical and laboratory data, such as hypersensitive C-reactive protein, erythrocyte sedimentation rate, CT, and biological factors [4], can facilitate the diagnosis of EPS. Moreover, we explored a new method—SWE-evaluated elastic values of the mesentery with histological reference.

Previous studies suggested that the duration of PD was the main risk factor for EPS [5]. Therefore, it was recommended that patients with long-term ambulatory PD undergo regular follow-up examinations. Although ultrasound is not as sensitive to calcification as CT, it can detect the thickening, stiffness, and calcification of the peritoneum and bowel wall in patients with long-term PD, which confirms its feasibility for routine follow-ups. Because it is easy to operate and non-radioactive, it is suitable for routine review of long-term PD patients. To date, the measurement of the normal peritoneum has not been established. In our previous study, we explored the SWE of patients with different peritoneal dialysis duration, namely, less than 3 months [5.20 kPa (IQR 3.10–7.60)], from 3 months to 5 years [6.40 kPa (IQR 4.10–10.5)], from 5 to 10 years [11.9 kPa (IQR 7.40–18.2)], and more than 10 years [19.3 kPa (IQR 11.7–27.3)] [6]. The information provided by SWE on tissue stiffness allows for better assessment of the peritoneum, and in combination with other examination methods, it can better guide patients to early intervention and efficient treatment strategies.

## Ethical approval

Written informed consent was obtained from the patients for publishing ultrasound and pathology images, and laboratory data after the de-identification of personal information.
